# Azolla incorporation under flooding reduces grain cadmium accumulation by decreasing soil redox potential

**DOI:** 10.1038/s41598-021-85648-x

**Published:** 2021-03-18

**Authors:** Chen Liu, Bin Guo, Hua Li, Qinglin Fu, Ningyu Li, Yicheng Lin, Guozhong Xu

**Affiliations:** 1 Institute of Environment, Resource, Soil and Fertilizer, Zhejiang Academy of Agricultural Sciences, Hangzhou, 310021 China; 2 Agricultural Ecology Institute, Fujian Academy of Agricultural Sciences, Fuzhou, 350003 China

**Keywords:** Microbiology, Environmental sciences

## Abstract

Cadmium (Cd) presents severe risks to human health and environments. The present study proposed a green option to reduce bioavailable Cd. Rice pot experiments were conducted under continuous flooding with three treatments (T1: intercropping azolla with rice; T2: incorporating azolla into soil before rice transplantation; CK: no azolla). The results showed that azolla incorporation reduced soluble Cd by 37% compared with the CK treatment, which may be explained by the decreased soil redox potential (Eh) (*r* = 0.867, *P* < 0.01). The higher relative abundance of *Methylobacter* observed in azolla incorporation treatment may account for dissolved organic carbon increase (*r* = 0.694; *P* < 0.05), and hence decreased the Cd availability for rice. Azolla incorporation increased the abundance of *Nitrospira*, indicating the potentially prominent role of nitrogen mineralization in increasing rice yields. Further, lower soluble Cd decreased the expression of *OsNramp5*, but increased *OsHMA3* levels in rice roots, which decreased Cd accumulation in grains. Through these effects, azolla incorporation decreased Cd concentrations in rice grains by 80.3% and increased the production by 13.4%. The negligible amount of Cd absorbed by azolla would not increase the risk of long-term application. Thus, intercropping azolla with early rice and incorporating azolla into soil before late rice transplantation can contribute to safe production at large scales of double rice cultivation.

## Introduction

Rice (*Oryza sativa* L.) is the most widely consumed staple food in China and other countries in Asia. However, it has a high absorptive efficiency for cadmium (Cd), which has led to high dietary Cd intake and poses serious threats to human health^1–3^. Various chemical and biological approaches have been proposed to control Cd uptake by rice plants in paddy fields^4^, such as lime, phosphate compounds, metal oxides and organic matter (OM) amendment^5,6^. However, these methods are still limited in practice, especially at large scales because of cultivation customs, regional disparities, costs, and potential secondary pollution risks. Therefore, a viable approach to decrease Cd bioavailability with cost-efficient and undisturbed cultivation habits still needs to be explored.

It is well documented that the soil redox potential (Eh) is the key factor in controlling Cd solubility and bioavailability^7,8^. In general, Eh has been controlled by water management. Under flooding, the Eh was approximately −200 mV which leads to reducing. Furthermore, dissolved Cd declines and is transformed into Cd-sulfide (CdS) or iron-manganese (oxyhydro) oxides with low solubility^9,10^. It has been widely accepted that Cd accumulation in rice increases during the heading and maturing stages, and is accompanied by a higher Eh caused by drainage^11,12^.

In a previous experiment, we found that the process of azolla incorporation could significantly decrease soil Eh after submergence. We hypothesized that the azolla incorporation could decrease Cd phytoavailability by regulating Eh. In addition, as a type of OM, azolla decomposition may reduce Cd mobility by forming complexes without increasing Cd bioavailability^6,13^. However, to our knowledge, few investigations have been conducted to study the remediation effects of azolla incorporation.

Soil bacterial community are highly sensitive to soil Eh^14,15^. Soil microbes affect plant metal accessibility through their involvement in the decomposition, stabilization and maintenance of soil physical and chemical conditions, such as *Proteobacteria* and *Acidobacteria*^16,17^. In addition, microbial community composition affectes plant growth and Cd accumulation by complex interactions^18^. Therefore, it is essential to clarify the effect of azolla incorporation on soil bacterial communities, which may in turn accelerate the reduction process and lead to ripple effects on Cd mobility.

Furthermore, the final grain Cd concentration of is controlled by several key processes, such as absorption by roots via *OsNramp5*, and root to shoot translocation by xylem via *OsHMA3*^19–21^. The gene expression of specific rice varieties is certain, and soil Cd mobility in soil may affect efficiency. For example, decreased *OsNramp5* levels and increased *OsHMA3* levels were present in upland rice/ *Solanum nigrum* intercropping systems, which induced lower DTPA-Cd concentrations^22^. It is not clear whether the reduced Cd uptake is caused by changing Cd transporters levels in rice, or by reducing the availability of soil Cd due to incorporation of a sculpted bacterial community.

Therefore, this study aimed to investigate the effects of azolla incorporation on plant growth, Cd accumulation, soil parameters and bacterial change in the rhizosphere, and the gene expression of related Cd transporters in rice under flooding. We hypothesized that the azolla incorporation (i) decreases rhizosphere Eh and soluble Cd, and changes the soil bacterial community, thereby decreasing Cd availability for rice; and (ii) decreases Cd concentration in various rice parts by downregulating Cd transporter genes in the roots and enhancing biomass. Our findings provide a green approach for reducing cadmium accumulation in rice, that can be applied at large-scales.

## Results

### Eh, pH, and Cd and SO_4_^2−^ concentration of soil solution

**Figure 1:**
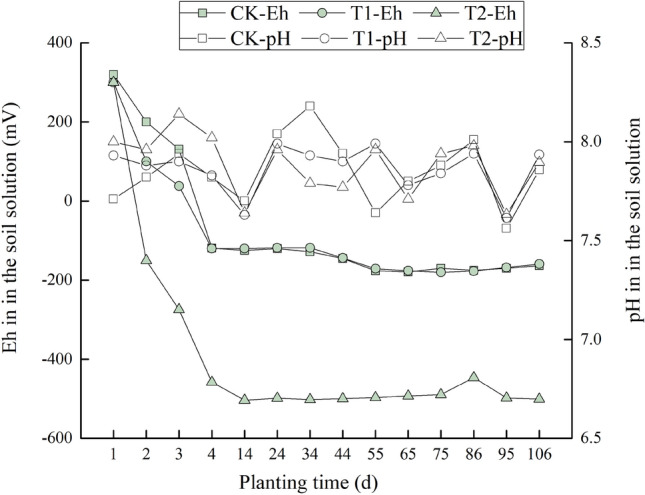
Changes in soil Eh and pH among different treatments. Treatments: no azolla (CK), azolla culturing in the water layer and intercropping with rice (T1), azolla incorporated into soil before rice transplantation (T2).

Soil Eh differed with the status of azolla (Fig. 1). With azolla incorporation (T2), Eh decreased significantly from 300 to −274 mV in the first 2 d and then remained stable at approximately −500 mV. The soil Eh of CK decreased from 300 to 131 mV in the first 2 d, which was slower than that of T2. The Eh of the azolla intercropping treatment (T1) remained approximately −170 mV, which was similar to that of CK. During the growth period, pH values fluctuated between 7.7 and 8.1. There were no significant pH differences among all treatments.

As shown in Table 1, the Cd concentrations in soil solutions of the azolla incorporation treatment (T2) were 0.11 μg L^−1^ and 0.10 μg L^−1^ at the tillering and maturing stages, respectively, which were significantly lower than those of CK. There were no differences in Cd concentrations between the CK and azolla intercropping treatments (T1). Compared with the CK treatment, azolla incorporation treatment (T2) decreased SO_4_^2-^ concentrations in the soil solutions by 54.5–72.9%. The SO_4_^2−^ concentrations in soil solution of the azolla intercropping treatment (T1) were similar to those of the CK treatment.

**Table 1:** Changes in Cd and SO_4_^2-^ concentrations in soil solutions at different growth stages.

Planting time (d)	Treatment	Cd / (μg L^−1^)	SO_4_^2−^ / (mg L^−1^)
2	CK	0.20 ± 0.03 a	790 ± 76.8 a
	T1	0.17 ± 0.01 a	722 ± 133 a
	T2	0.18 ± 0.02 a	359 ± 104 b
35 tillering stage	CK	0.17 ± 0.02 a	572 ± 108 a
	T1	0.16 ± 0.03 a	626 ± 145 a
	T2	0.11 ± 0.01 b	156 ± 73.7 b
106 maturing stage	CK	0.16 ± 0.02 a	454 ± 99.1 a
	T1	0.14 ± 0.01 a	448 ± 104 a
	T2	0.10 ± 0.01 b	123 ± 57.4 b

Treatments: no azolla (CK), azolla culturing in the water layer and intercropping with rice (T1), azolla incorporated into soil before rice transplantation (T2). Different letters represented significant difference at *P* < 0.05.

### Soil chemical properties

As shown in Table 2, azolla incorporation treatment (T2) significantly increased SOC, NH_4_^+^-N and NO_3_^–^N. Total nitrogen and total carbon were similar among all treatments. Compared with the CK treatment, azolla incorporation treatment (T2) decreased available Cd (–CaCl_2_) by 23.7%. There was a significant correlation between soluble Cd and available Cd (*r* = 0.784, *P* < 0.01).

**Table 2:** Soil physicochemical properties under different treatments.

Treatment	SOC/(mg kg^-1^)	TOC/(g kg^-1^)	NH_4_^+^–N/(mg kg^-1^)	NO_3_^–^N/(mg kg^-1^)	TN/(g kg^-1^)	CaCl_2_–Cd/(mg kg^-1^)
CK	33.3 ± 12.4 b	10.2 ± 0.23 a	3.46 ± 0.22 b	2.19 ± 0.27 b	1.76 ± 0.03 a	188 ± 8.13 a
T1	40.3 ± 7.97 b	10.4 ± 0.09 a	3.19 ± 0.09 b	1.84 ± 0. 14 b	1.80 ± 0. 09 a	198 ± 5.09 a
T2	74.5 ± 4.04 a	10.4 ± 0.31 a	4.12 ± 0.42 a	2.83 ± 0.39 a	1.80 ± 0.09 a	144 ± 11.4 b

Different letters represented significant difference at *P* < 0.05.

### Soil bacterial community

The bacterial community diversity indices are shown in Table S1. There were no significant differences in the Chao1, Simpson, Shannon indexes or in Good’s coverage.

**Figure 2:**
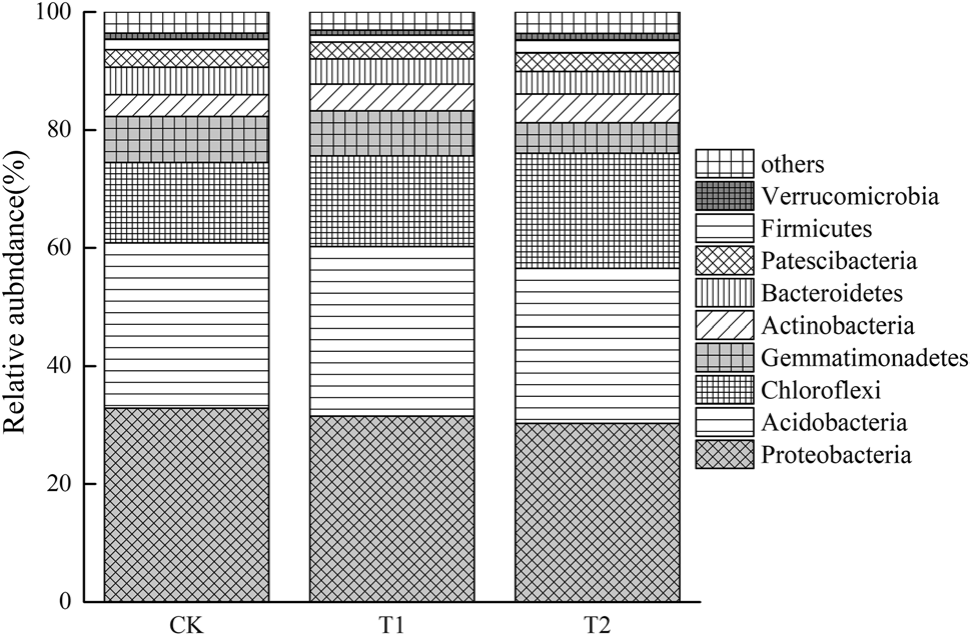
Relative abundances (> 1%) of different bacterial phyla.

Eight dominant phyla (relative abundance > 1%) included *Proteobacteria* (~ 32%), *Acidobacteria* (~ 28%), *Chloroflexi* (~ 16%), *Gemmatimonadetes* (~ 7%), *Actinobacteria*, *Bacteroidetes*, *Patescibacteria*, *Firmicutes*, and *Verrucomicrobia* (Fig. 2). The ranked order of these phyla was similar across all treatments, except for *Chloroflexi* and *Gemmatimonadetes*. Compared with the CK treatment, azolla incorporation increased the relative abundances of *Chloroflexi* by 43.4% but decreased the relative abundances of *Gemmatimonadetes* by 33.7%.

**Figure 3:**
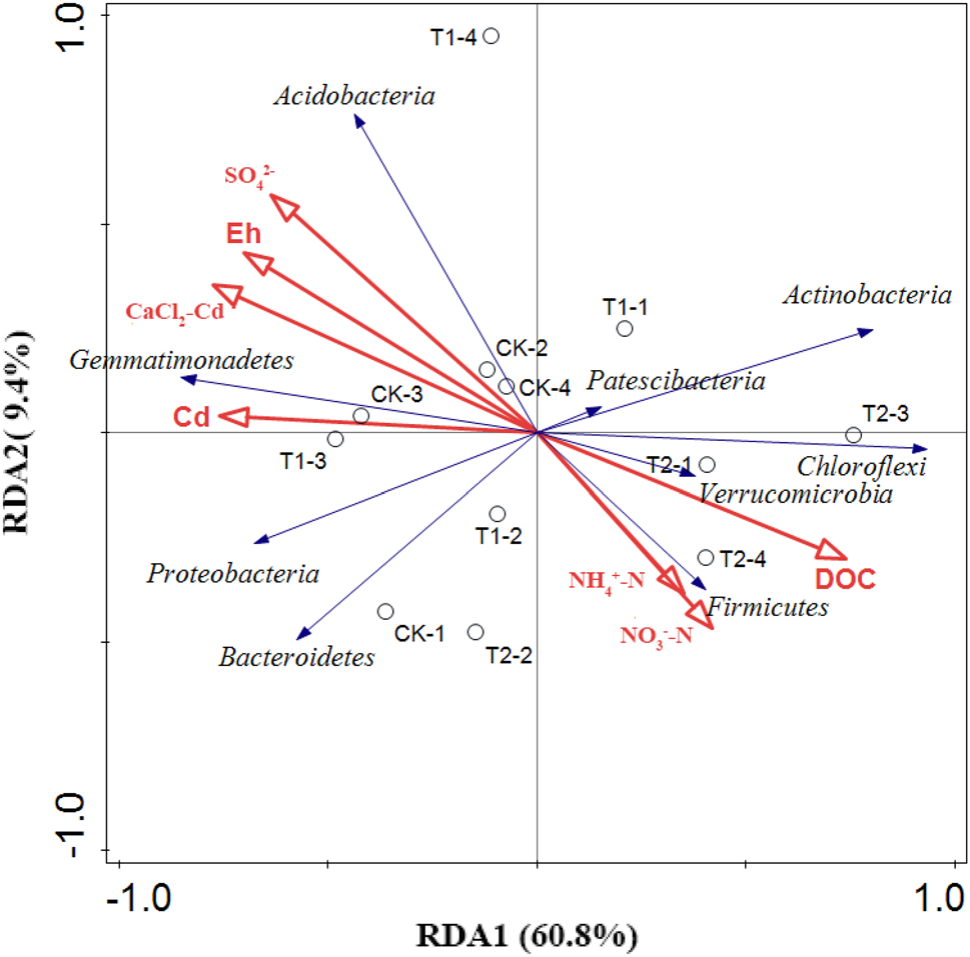
RDA between soil parameters and soil bacterial structure at the phylum level.

Redundancy analysis (RDA) explained 70.2% of the variance, and it appears that azolla incorporation affected the structure of the soil bacterial community (Fig. 3). The azolla incorporation treatment (T2) had a close relationship with the relative abundances of *Chloroflexi*, and the CK treatment had a close relationship with the relative abundances of *Gemmatimonadetes*. Among all environmental variables, Cd concentration in soil solution was the most important factor influencing rhizosphere bacterial communities (*P* < 0.05). According to the results of correlation analysis, the relative abundances of *Chloroflexi* had a significant negative correlation with the Eh, and SO_4_^2-^, Cd concentrations in the soil solutions (*r* = −0.688, *r* = -0.623, *r* = −0.726; *P* < 0.05, respectively), while the relative abundances of *Gemmatimonadetes* had a significant positive correlation (*r* = 0.754, *r* = 0.699, *r* = 0.751; *P* < 0.05, respectively).

**Figure 4:**
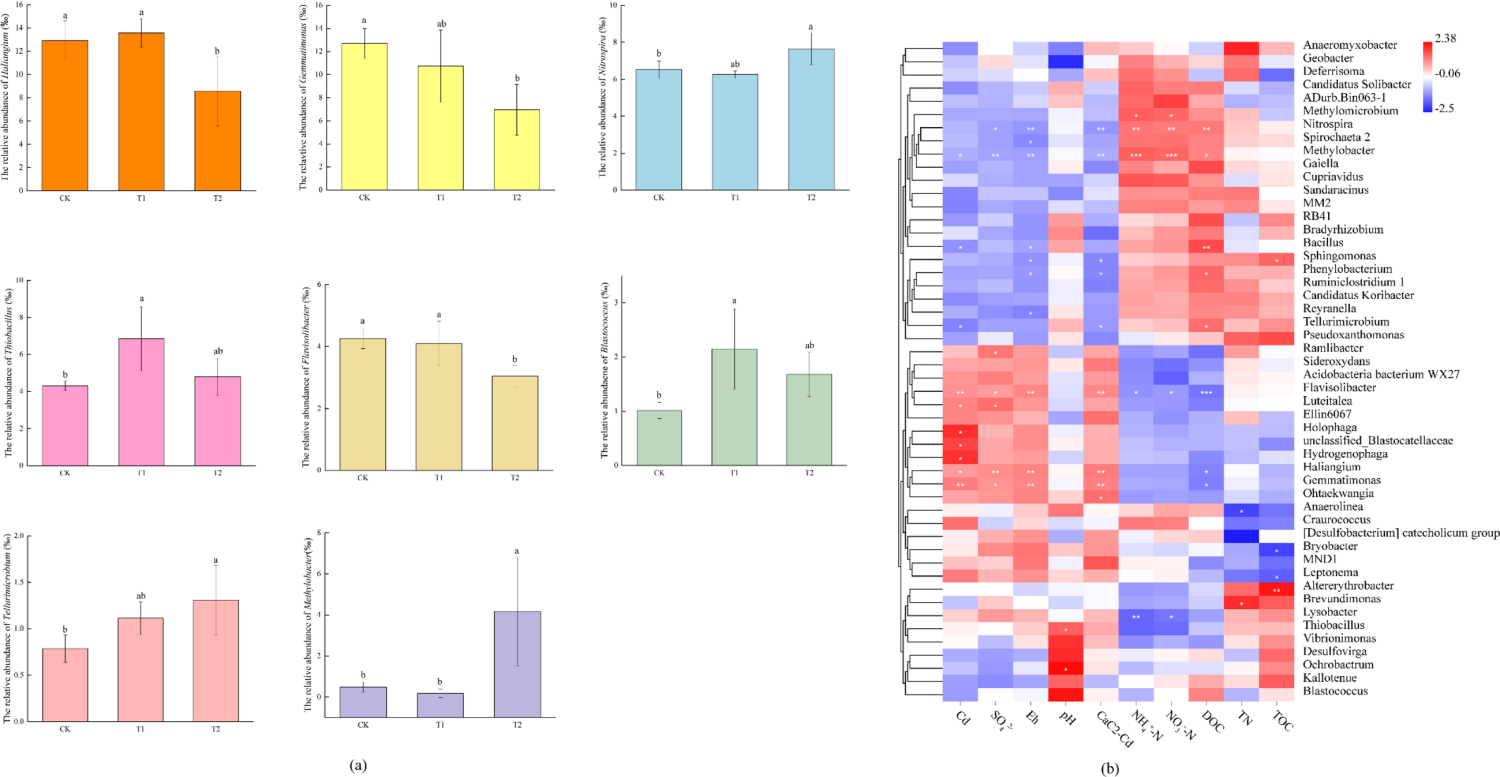
(**a**) Relative abundance of different genus under different treatments. Different letters represented significant difference at *P* < 0.05. (**b**) Pearson correlations on soil properties and the top 50 genus. Blue and red represent negative and positive correlations, respectively, with darker colors representing higher correlations. *, indicate a significant difference at the level of *P* < 0.05, **, indicate a significant difference at the level of *P* < 0.01.

The 50 most abundant genus of the 12 samples indicated a clear difference in bacterial community structure among all treatments (Fig. 4). Azolla incorporation resulted in lower abundances of *Haliangium* (*Proteobacteria*), *Gemmatimonas* (*Gemmatimonadetes*), and *Flavisolibacter* (*Bacteroidia*), which have a positive relationship with soil Cd and Eh (Table S2). The relative abundances of *Methylobacter* (*Gammaproteobacteria*) and *Nitrospira* (*Nitrospirae*) were higher in the azolla the azolla incorporation treatment (T2) and had positive correlations with dissolve organic carbon (DOC), NH_4_^+^-N and NO_3_^–^N.

### Relative expression of *OsNramp5 *and *OsHMA3* in roots

**Figure 5:**
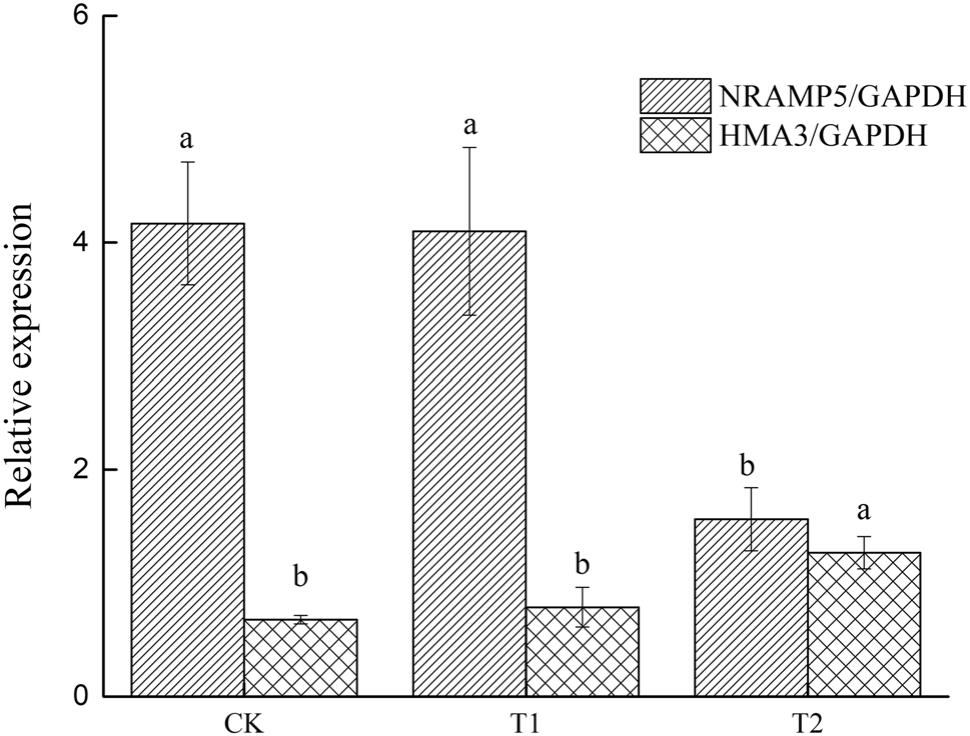
Relative expression of *OsNramp5* and *OsHMA3* genes in roots. Different letters represented significant difference at *P* < 0.05.

Compared with the CK treatment, the relative expression of *OsNramp5* in roots was significantly lower in the azolla incorporation treatment (T2), in which the Cd concentration were lower in roots (Fig. 5). In addition, the expression level of *OsNramp5* was correlated with the total Cd content in roots (*r* = 0.81, *P* < 0.01). Compared with the CK treatment, azolla incorporation treatment (T2) upregulated *OsHMA3* expression, which resulted in less Cd accumulation in grains. The translocation factor was also lower with the azolla incorporation treatment (T2) than with the CK treatment. The expression levels of *OsHMA3* were significantly negatively correlated with TF’ (*r* = −0.75, *P* < 0.01).

### Cd concentration in rice and azolla

Under alone flooding (CK) without azolla, the Cd accumulations in roots, straw and grains were 14.6, 1.07 and 0.46 mg kg^-1^, respectively (Table 3). The Cd concentrations in roots with the azolla incorporation treatment (T2) decreased significantly by 49.8% compared with the CK treatment. Furthermore, Cd concentrations in grains of the azolla incorporation treatment (T2) were only 0.09 mg kg^-1^, which were lower than those of CK (0.46 mg kg^-1^). Cd concentrations in grains were similar between T1 and CK, which were consistent with the results of similar Eh and Cd concentrations in the soil solutions. Cd concentrations in roots, straw and grains were positively correlated with Cd concentrations in soil solutions (roots: *r* = 0.90; straw: *r* = 0.78; grains: *r* = 0.89; *P* < 0.01). Compared with the CK treatment, azolla incorporation decreased the TF’ by 56.4%, which indicated lower Cd transport from roots to shoots due to the decreased amount of Cd absorption by roots. The Cd concentration and content of azolla in azolla intercropping treatment (T1) were 0.90 mg kg^-1^ and 4.84 μg pot^-1^, respectively, which were much lower than those in rice (30.2–69.6 μg) (Table S3). Owing to the low Cd content of azolla, azolla incorporation process would not increase soil Cd bioavailability.

**Table 3:** Cd concentrations in the roots, straw and grains of rice and its translocation factor from roots to shoots.

Treatment	Grains	Straw	Roots	TF’
	Cd concentration /mg L^−1^			
CK	0.46 ± 0.03 a	1.07 ± 0.10 a	14.6 ± 2.70 a	0.78 ± 0.19 a
T1	0.40 ± 0.08 a	1.29 ± 0.23 a	14.3 ± 0.91 a	0.82 ± 0.16 a
T2	0.09 ± 0.04 c	0.27 ± 0.08 b	7.35 ± 0.92 b	0.34 ± 0.08 b

Different letters represented significant difference at *P* < 0.05.

**Figure 6:**
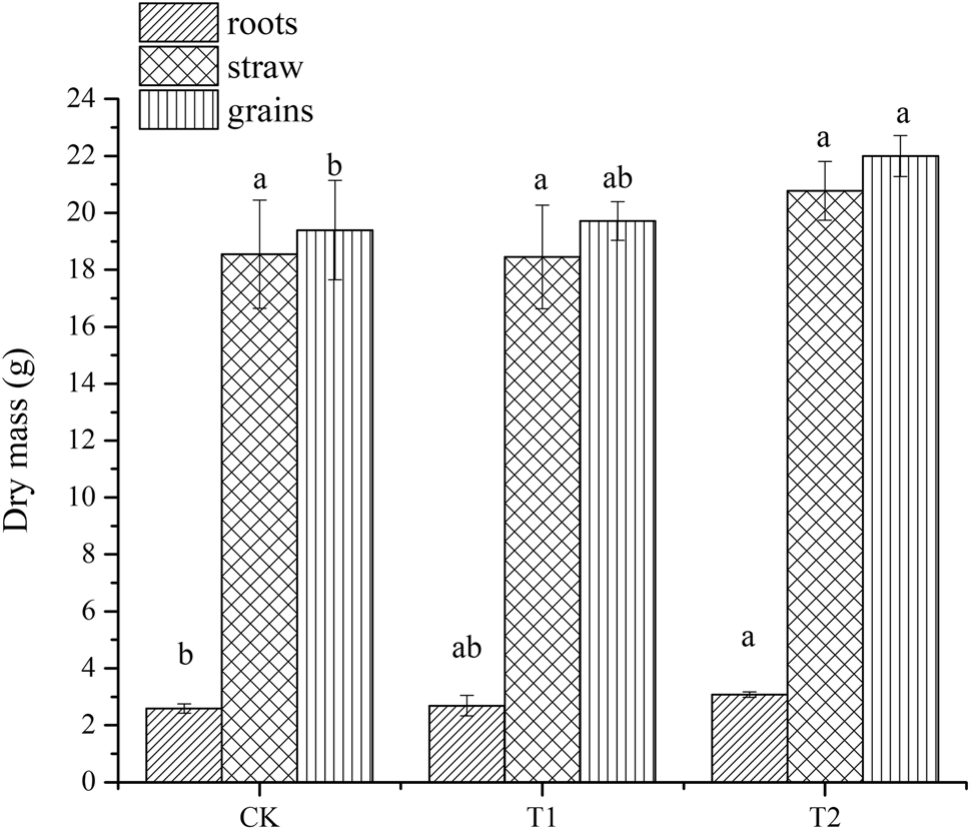
Roots, straw, and grains dry masses of rice. Different letters represented significant difference at *P* < 0.05.

The azolla incorporation treatment enhanced the growth of rice. Compared with the CK treatment, the dry mass of roots, straw and grains increased by 18.7%, 12.1% and 13.4%, respectively (Fig. 6).

## Discussion

In recent years, many in-situ stabilization techniques have been tested on Cd-contaminated paddy soils to reduce uptake by rice^5^. In our study, azolla incorporation led to a significant decrease in soil soluble Cd, which may be due to decreased Eh and SO_4_^2-^concentrations (*r* = 0.83 and *r* = 0.88, respectively; *P* < 0.01). Previous studies have verified that Cd mobility could decrease at lower Eh levels^8,23^. Since sulfate reduction occurs under reducing situations, poorly soluble CdS is formed, which results in a reduced Cd mobility^10,24,25^. As Eh increased, sulfate concentrations in soil solutions increased by oxidizing sulfur, and forming soluble CdSO_4_^26,27^. Another possible explanation is that when incorporated into soil, a considerable amount of organic C can be supplied, which significantly increase soil DOC levels. The increased levels of DOC and solid organic matter could decrease Cd phytoavailability by forming complex and increasing surface charge^6,13^. Increased OM may also enhance sulfate reduction and decrease Cd mobility^28^. In general, soil pH is an important factor that affects Cd phytoavailability with negative correlation^6,29^. In our present study, pH levels increased from 5.8 to approximately 8.0 once flooded. However, pH values were similar among treatments, which were inconsistent with Cd concentrations in the soil solutions. Thus, pH is not the main reason to account for the lower soluble Cd level in azolla incorporation treatment.

At the phylum level, the orders of primary bacteria and microbial α diversity in the rhizosphere soils of all treatments were similar^15,30^. This is likely due to community readjustment, as some metal-sensitive bacteria increase, and some resistant bacteria decrease as Cd concentrations change. In our study, azolla incorporation treatment decreased the relative abundance of *Gemmatimonadetes*. An et al*.* reported that *Gemmatimonadetes* was higher in contaminated soil with a high tolerance to Cd stress^31^. At genus level, *Flavisolibacter* (*Bacteroidetes*) was higher in the CK treatment with higher Cd concentration. A previous study also reported that Cd can stimulate the abundance of *Flavisolibacter*, which could catalyze hydrogen peroxide under high Cd contamination^32^. Specific species involved in Cd immobilization were modified after azolla incorporation. It has been reported that increased abundances of *Chloroflexi* were involved in immobilizing soil Cd^33^. *Chloroflexi* are photosynthetic bacteria that affect the stability of microflora/fauna communities by involving the cycling of iron and manganese^34^.

As a kind of methane-oxidizing bacteria, *Methylobacter* (*Gammaproteobacteria*) prefers oxygen-deficient conditions and attributes to anaerobic oxidation of methane with interconnection between denitrifiers and iron-cycling partners^35^. In our study, the higher relative abundance of *Methylobacter* in azolla incorporation treatment is positively correlated with DOC (*r* = 0.694, *P* < 0.05). Studies confirmed that organic residues addition significantly increased the soil methane production potentials and DOC level^36,37^. Straw input could also stimulate soil nitrite oxidizing potential by affecting the relative abundance of *Nitrospira*, which is reported to be a major players in nitrite oxidation^38,39^. Incorporating azolla into soil could increase rice yield and soil fertility by providing additional N nutrient^40^. This could explain why azolla incorporation treatment (T2) had the higher content of available N, which were beneficial to increase yields.

In our study, the low Cd bioavailability with azolla incorporation induced decreased root-to-shoot translocation. Zhang et al*.* also demonstrated that root-to-shoot translocation of Cd decreased under flooding^14^. These results were also confirmed by the gene expression results of *OsNramp5* and *OsHMA3*. Downregulation of *OsNramp5* suppressed Cd transport from soil solutions into root cells, which reduced root Cd accumulation^41^. Upregulated *OsHMA3* could reduce shoot Cd accumulation but sequester more Cd in root cells^19,42^. Possible mechanism is that lower Cd concentrations in the rhizosphere directly trigger and regulate the expression of related genes in rice roots. For example, compared with monoculture, decreased *OsNramp5* levels and increased *OsHMA3* expression were present in upland rice/*Solanum nigrum* intercropping systems, which induced lower DTPA-Cd concentrations^22^. Similar results were found by Chen et al.^43^.

Cd concentrations in rice were similar between T1 and CK, which confirmed that azolla intercropping did not influence Cd accumulation in rice. Compared with the single flooding control, Cd concentrations in rice (roots, straw, and grains) declined dramatically by 49.8–295% with azolla incorporation. Notably, Cd concentrations in grains were lower than the Chinese national standard of 0.20 mg kg^−1^ under such high soil Cd contamination levels (1.12 mg kg^-1^). The results indicated that incorporating azolla into soil could dramatically decrease Cd accumulations in rice. This effect is even better than that of limestone, a popular practice that decreases Cd in grains (~ 0.3 mg kg^-1^) without affecting yield under similar contamination levels^44^. Total Cd concentration and bioavailable Cd fractions of organic amendments determined the long-term effects on Cd immobilization. Cd concentration in the soil surface and water layer is 4–6 times lower than in deep soil solution, the Cd absorbed by azolla was negligible (Table S2). This unique characteristic makes it as a ‘clean’ OM, different from straw or other green manure. Thus, azolla incorporation under flooding can be recommended as a practical method to control Cd bioavailability at large scales of double rice cultivation.

## Conclusions and prospects

In the present study, we proposed azolla incorporation combined with water management as a green approach to decrease Cd accumulation in rice grains. Soluble Cd decrease was achieved because of greater Eh decrease. Less phytoavailable Cd could be caused by the higher relative abundance of *Methylobacter* that involved in carbon mineralization. Additionally, azolla incorporation increased the relative abundance of *Nitrospira* and affected the N dynamics. Decreasing Cd bioavailability reduces the translocation and accumulation of Cd in grains by changing the expression of *OsNramp5* and *OsHMA3*. Taken together, azolla incorporation result in a Cd accumulation reduction in grains. This finding provided a feasible and economical strategy to meet the requirements of Cd reduction and production promotion, which could be widely used at large-scale polluted double rice fields. Azolla is intercropped with early rice as a dual crop and then incorporated into soil before late rice transplanting. It should be noted that the effects are practical-dependents. Further field experiments under multi season are also needed.

## Materials and methods

### Soil properties and pot experiment.

Paddy soil for the pot experiment was collected from Linan (China), which has a textural composition of 52% sand, 30% silt and 18% clay. The parent material of this soil was fluvial deposits. Soil organic carbon (SOC) and total nitrogen (TN) content were 9.87 g kg^-1^ and 1.32 g kg^-1^, respectively. Total Cd and 0.1 M CaCl_2_-extractable Cd concentrations were 1.12 and 0.31 mg kg^-1^, respectively. Soil pH was 5.80 (1:2.5 soil/water). CEC was 15.28 *cmol* kg^-1^. Each treatment consisted of four replicated pots each containing 10 kg of soil and with mixing fertilizers (N, P and K) at 0.5 g pot^-1^. Urea (0.2 g pot^-1^) was topdressed at 10 and 60 d after transplantation.

The treatments included (1) no azolla as the control, CK; (2) 50 g of fresh azolla (*Azolla caroliniana willd*, provided by Agricultural Ecology Institute, Fujian Academy of Agricultural Sciences) cultured in the water layer, intercropping with rice, T1; and (3) 200 g of fresh azolla incorporated before rice cultivation, incorporated into soil before rice transplanting, T2 (Fig. S1). After propagating for about 10 days, the amount of azolla in treatment T1 was about 200 g. Total carbon and nitrogen of azolla were 41.5% and 2.64%, respectively. All pot was flooded during the entire rice growth season with at least a 3 cm water layer on the soil surface. If the water layer fell below the minimum limit, we irrigated gently to avoid disturbing the soil layer.

Rice seeds (*Oryza sativa* L. cv. ZheFuJing83) were sterilized with 5% sodium hypochlorite for 15 min and washed extensively with sterilized water. These seeds were then incubated at 30 ℃ for 48 h in darkness and were geminated in wetted filter paper at 35 ℃ for an additional 24 h. Finally, uniformly germinated seeds were selected and transferred to pots after four weeks. Each pot contained three plants. Plants were grown in a greenhouse and was irrigated with tap water. Rice was transplanted on July 17 and harvest on October 30.

### Sampling

Soil Eh was measured at 10 cm beneath the soil surface using an Eh meter and platinum electrodes in each pot. Soil pH was also measured in situ with a pH electrode. Except for the first 3 d, soil Eh and pH values were recorded at 10 d intervals. A clay pot end soil solution extractor (suction-cup method) was adopted for in-situ sampling. It was inserted in each plot into the 10 cm soil layer before transplantation. When sampling, water in the tube was drained in advance, and 20 mL of soil solution was then extracted by a syringe 1 h later. The samples were passed through a 0.2 μm filter. The filtered samples were used for dissolved Cd and SO_4_^2-^ analysis. At the maturing stage (October 30), roots, straw and grains were separated and air-dried for analysis. Soil samples, that tightly adhered to the rice roots were collected to determine soil chemical properties and bacterial community structures.

### Chemical analyses

For biomass measurements, each part of the rice plant was washed and dried (at 105 ℃ for 0.5 h and at 70 ℃ for 3 days).

After the soil solution was filtered, dissolved Cd and SO_4_^2-^ levels were measured using plasma mass spectrometry and ion chromatography (861, Metrohm, Switzerland), respectively.

SOC and TN were determined by using the element analyzer (vario ISOTOPE CUBE, Elementar, Germany). DOC was calculated as mg DOC/kg soil (H_2_O extraction method, Multi N/C 3100, Analytikjena, Germany)^45^. Concentrations of NH_4_^+^–N and NO_3_^–^-N were measured using a segmented-continuous flow analyzer (AutoAnalyzer 3, Bran + LUEBBE, Germany).

Plant Cd concentrations were determined using the procedure reported by Guo et al. via plasma mass spectrometry (PlasmaQuant MS Elite, Analytikjena, Germany)^46^. Calibration was applied using seven standards (e.g., 0.1, 0.2, 0.5, 1, 2, 5 and 10 μg kg^−1^). Two additional calibration standards (e.g., laver composition GBW10023, 0.57 mg kg^−1^; soil composition GBW07456, 0.59 mg kg^−1^) served as quality control samples for plants and soil during the plant measurement sessions. Mass fractions were calculated using linear regression equation. The obtained mass fractions for trace metals were further corrected for the respective procedural blank and recovery rate.

### Real-time RT-PCR analysis of *OsNramp5* and *OsHMA3* expression in roots

The amplification procedure for *OsNramp5*, *OsHMA3* and GAPDH (glyceraldehyde 3-phosphate dehydrogenase, as an internal control) was described by Chen et al.^43^. Details of the primers used are shown in Table S4. Each sample was run in three technical replicates. The relative expression of *OsNramp5* and *OsHMA3* was determined using the 2^–ΔΔCT^ method (ABI Step One, Applied Biosystems, USA)^47^.

### MiSeq

For each pot, three soil subsamples were collected. Total DNA in soil was extracted using an E.Z.N.A.Soil DNA Kit (Omega, USA). The V3-V4 region of the 16S rRNA gene were amplify using primers 338F (5′- ACTCCTACGGGAGGCAGCA-3′) and 806R (5′- GGACTACHVGGGTWTCTAAT-3′)^48^. Sequencing was conducted at the Shanghai Personal Biotechnology Co., Ltd (Shanghai, China) using the Illumina MiSeq platform with a MiSeq Reagent Kit v3. A 97% similarity of the obtained sequences was used as the criterion for the classifying of the operational taxonomic units (OTU). Alpha and beta diversities were determined using the QIIME and R packages (v3.2.0). Taxa abundances were statistically compared among samples using Metastats. A heat map was generated to show the top 50 differentially abundant genera. Principal coordinate analysis (PCoA) was performed using unweighted UniFrac distance metrics.

### Statistical analysis

The translocation factor from metal accumulation (TF’) was calculated as the ratio between the Cd accumulation content (mg) in aboveground plants (straw and grains) and roots. ANOVA followed by Tukey’s test and correlation analysis were performed using SPSS v22.0. Significance was determined at the *P* < 0.05 level. Redundancy analysis (RDA) was adopted to assess the effects of soil chemical parameters on soil microbial diversity using Canoco 5. Spearman correlation coefficients were employed to test relationships between soil properties and the relative abundance of genera using the OmicShare tools (http://www.omicshare.com/tools). All values are presented as means ± standard deviation (s.d.).

## Plant material statement

The plants adopted in this study are not threatened species or wild plants. Fresh azolla was provided by Agricultural Ecology Institute, Fujian Academy of Agricultural Sciences. Rice seeds (ZheFuJing83) were bought from market, which is a conventional rice variety in southern China. All procedure complies with relevant institutional, national, and international guidelines and legislation.

## Supplementary Information

Supplementary Information
