# Supplementary material for: Azolla incorporation under flooding reduces grain cadmium accumulation by decreasing soil redox potential
Source: Sci Rep. 2021 Mar 18;11:6325. doi: 10.1038/s41598-021-85648-x (PMC7973551; doi:10.1038/s41598-021-85648-x)
Supplement: Supplementary file 1 — Supplementary Information [file 41598_2021_85648_MOESM1_ESM.docx]

**Azolla incorporation under flooding reduces grain cadmium accumulation by decreasing soil redox potential**

Chen Liu^1^, Bin Guo^1^, Hua Li^1^, Qinglin Fu^1^, Ningyu Li^1^, Yicheng Lin^1*^, Guozhong Xu^2^

1. Institute of Environment, Resource, Soil and Fertilizer, Zhejiang Academy of Agricultural Sciences, Hangzhou 310021, China

2. Agricultural Ecology Institute, Fujian Academy of Agricultural Sciences, Fuzhou 350003, China

*Corresponding author: Yicheng Lin, associate professor, +86 571 86404041, lyc5918@sina.com

Table S1 The diversity indices of all treatments.

| Treatment | Chao1 | Simpson | Shannon | Goods coverage |
| --- | --- | --- | --- | --- |
| CK | 3918±382 | 0.99±0.01 | 9.84±0.29 | 0.99±0.01 |
| T1 | 3977±299 | 0.99±0.01 | 9.70±0.16 | 0.99±0.01 |
| T2 | 4130±548 | 0.99±0.01 | 9.64±0.24 | 0.99±0.01 |

Table S2 Correlation analysis between microbial community and soil parameters at genus level.

|  |  | Cd | SO_4_^2-^ | Eh | pH | CaCl_2-_Cd | NH_4_^+^-N | NO_3_^-^-N | DOC | TN | TOC |
| --- | --- | --- | --- | --- | --- | --- | --- | --- | --- | --- | --- |
| *Anaeromyxobacter* | *r* | -.215 | .024 | -.241 | .031 | -.078 | .155 | .031 | -.078 | .440 | .156 |
|  | *P* | .502 | .941 | .451 | .925 | .810 | .631 | .925 | .810 | .152 | .628 |
| *Bryobacter* | *r* | .029 | .323 | .134 | -.180 | -.068 | .291 | -.180 | -.068 | -.332 | -.664^*^ |
|  | *P* | .929 | .306 | .678 | .576 | .833 | .359 | .576 | .833 | .292 | .019 |
| *Ramlibacter* | *r* | .307 | .576^*^ | -.150 | -.279 | -.477 | .405 | -.279 | -.477 | .440 | .062 |
|  | *P* | .331 | .050 | .642 | .380 | .117 | .192 | .380 | .117 | .152 | .849 |
| *Ellin6067* | *r* | .207 | .186 | -.156 | -.193 | -.138 | .243 | -.193 | -.138 | .110 | -.114 |
|  | *P* | .518 | .563 | .628 | .547 | .669 | .446 | .547 | .669 | .735 | .724 |
| *MND1* | *r* | .094 | .072 | -.051 | .004 | -.264 | .306 | .004 | -.264 | -.193 | -.319 |
|  | *P* | .770 | .823 | .874 | .990 | .407 | .334 | .990 | .407 | .548 | .311 |
| *Candidatus Solibacter* | *r* | -.350 | -.238 | .169 | .290 | .297 | -.171 | .290 | .297 | -.134 | -.254 |
|  | *P* | .265 | .456 | .601 | .361 | .348 | .594 | .361 | .348 | .679 | .426 |
| *Geobacter* | *r* | -.164 | .036 | -.474 | .117 | .047 | -.053 | .117 | .047 | .226 | -.084 |
|  | *P* | .610 | .912 | .119 | .717 | .886 | .871 | .717 | .886 | .480 | .795 |
| *Haliangium* | *r* | .640^*^ | .716^**^ | .131 | -.496 | -.672^*^ | .805^**^ | -.496 | -.672^*^ | .053 | -.373 |
|  | *P* | .025 | .009 | .685 | .101 | .017 | .002 | .101 | .017 | .871 | .233 |
| *Gemmatimonas* | *r* | .757^**^ | .647^*^ | -.007 | -.456 | -.688^*^ | .741^**^ | -.456 | -.688^*^ | -.041 | -.332 |
|  | *P* | .004 | .023 | .982 | .136 | .013 | .006 | .136 | .013 | .899 | .292 |
| *Sphingomonas* | *r* | -.397 | -.447 | -.109 | .247 | .467 | -.632^*^ | .247 | .467 | .484 | .667^*^ |
|  | *P* | .201 | .145 | .737 | .439 | .126 | .028 | .439 | .126 | .111 | .018 |
| *Nitrospira* | *r* | -.557 | -.688^*^ | -.151 | .712^**^ | .726^**^ | -.764^**^ | .712^**^ | .726^**^ | .244 | .033 |
|  | *P* | .060 | .013 | .640 | .009 | .008 | .004 | .009 | .008 | .444 | .918 |
| *Anaerolinea* | *r* | -.046 | -.138 | .253 | .129 | .104 | -.071 | .129 | .104 | -.610^*^ | -.481 |
|  | *P* | .887 | .668 | .427 | .689 | .748 | .828 | .689 | .748 | .035 | .113 |
| *Thiobacillus* | *r* | .116 | .078 | .642^*^ | -.498 | -.151 | .370 | -.498 | -.151 | .297 | .315 |
|  | *P* | .718 | .810 | .024 | .100 | .639 | .237 | .100 | .639 | .348 | .319 |
| *Sideroxydans* | *r* | .403 | .428 | .152 | -.519 | -.383 | .567 | -.519 | -.383 | .175 | -.004 |
|  | *P* | .193 | .166 | .637 | .084 | .219 | .055 | .084 | .219 | .587 | .991 |
| *Phenylobacterium* | *r* | -.554 | -.544 | -.064 | .392 | .622^*^ | -.707^*^ | .392 | .622^*^ | .269 | .291 |
|  | *P* | .062 | .067 | .844 | .207 | .031 | .010 | .207 | .031 | .397 | .359 |
| *Ohtaekwangia* | *r* | .402 | .456 | .209 | -.409 | -.441 | .621^*^ | -.409 | -.441 | -.169 | -.355 |
|  | *P* | .196 | .136 | .515 | .186 | .151 | .031 | .186 | .151 | .599 | .257 |
| *Candidatus Koribacter* | *r* | -.516 | -.430 | -.109 | .275 | .427 | -.430 | .275 | .427 | .437 | .276 |
|  | *P* | .086 | .163 | .736 | .388 | .167 | .162 | .388 | .167 | .156 | .385 |
| *Gaiella* | *r* | -.400 | -.324 | .009 | .241 | .559 | -.475 | .241 | .559 | .082 | .025 |
|  | *P* | .197 | .304 | .977 | .450 | .059 | .119 | .450 | .059 | .800 | .939 |
| *Flavisolibacter* | *r* | .709^**^ | .650^*^ | -.173 | -.632^*^ | -.865^**^ | .763^**^ | -.632^*^ | -.865^**^ | .126 | .083 |
|  | *P* | .010 | .022 | .590 | .027 | .000 | .004 | .027 | .000 | .696 | .797 |
| *MM2* | *r* | -.529 | -.378 | -.171 | .419 | .314 | -.292 | .419 | .314 | .380 | .052 |
|  | *P* | .077 | .226 | .596 | .175 | .320 | .357 | .175 | .320 | .224 | .871 |
| *ADurb.Bin063_1* | *r* | -.320 | -.363 | .121 | .562 | .204 | -.337 | .562 | .204 | -.367 | -.317 |
|  | *P* | .310 | .246 | .708 | .057 | .525 | .284 | .057 | .525 | .241 | .316 |
| *Ruminiclostridium_1* | *r* | -.394 | -.323 | -.114 | .320 | .529 | -.559 | .320 | .529 | .383 | .157 |
|  | *P* | .205 | .305 | .724 | .311 | .077 | .059 | .311 | .077 | .220 | .626 |
| *Desulfovirga* | *r* | -.192 | -.227 | .391 | .002 | .044 | .016 | .002 | .044 | -.152 | .265 |
|  | *P* | .549 | .477 | .209 | .994 | .893 | .961 | .994 | .893 | .637 | .405 |
| *Holophaga* | *r* | .675^*^ | .246 | -.060 | -.312 | -.256 | .256 | -.312 | -.256 | -.216 | -.235 |
|  | *P* | .016 | .442 | .853 | .324 | .421 | .422 | .324 | .421 | .500 | .462 |
| *RB41* | *r* | -.395 | -.239 | .181 | .065 | .388 | -.333 | .065 | .388 | -.264 | .229 |
|  | *P* | .203 | .455 | .573 | .841 | .212 | .290 | .841 | .212 | .406 | .474 |
| *Spirochaeta_2* | *r* | -.369 | -.486 | -.193 | .488 | .423 | -.470 | .488 | .423 | .124 | .074 |
|  | *P* | .238 | .109 | .548 | .107 | .171 | .124 | .107 | .171 | .702 | .818 |
| *Deferrisoma* | *r* | -.062 | -.035 | -.136 | .220 | -.081 | .138 | .220 | -.081 | .288 | -.245 |
|  | *P* | .848 | .914 | .673 | .491 | .804 | .668 | .491 | .804 | .364 | .443 |
| *Methylomicrobium* | *r* | -.446 | -.440 | -.113 | .607^*^ | .431 | -.348 | .607^*^ | .431 | .221 | -.305 |
|  | *P* | .146 | .152 | .726 | .036 | .162 | .267 | .036 | .162 | .491 | .335 |
| *Reyranella* | *r* | -.388 | -.524 | .114 | .373 | .506 | -.468 | .373 | .506 | .484 | .318 |
|  | *P* | .212 | .080 | .724 | .233 | .093 | .125 | .233 | .093 | .111 | .313 |
| *Bacillus* | *r* | -.671^*^ | -.460 | .298 | .380 | .709^**^ | -.555 | .380 | .709^**^ | -.253 | -.128 |
|  | *P* | .017 | .132 | .347 | .223 | .010 | .061 | .223 | .010 | .428 | .692 |
| *Desulfobacterium* | *r* | -.100 | -.016 | -.250 | -.073 | -.056 | .013 | -.073 | -.056 | -.468 | -.125 |
|  | *P* | .757 | .960 | .432 | .822 | .863 | .968 | .822 | .863 | .125 | .699 |
| *Ochrobactrum* | *r* | -.120 | -.225 | .576^*^ | -.048 | -.048 | .093 | -.048 | -.048 | .054 | .296 |
|  | *P* | .711 | .482 | .050 | .882 | .882 | .774 | .882 | .882 | .869 | .351 |
| *Sandaracinus* | *r* | -.454 | -.270 | -.443 | .193 | .243 | -.186 | .193 | .243 | .253 | -.098 |
|  | *P* | .138 | .397 | .149 | .548 | .447 | .563 | .548 | .447 | .427 | .762 |
| *Craurococcus* | *r* | .296 | -.175 | -.148 | .255 | -.049 | -.084 | .255 | -.049 | -.415 | -.381 |
|  | *P* | .351 | .586 | .647 | .423 | .881 | .796 | .423 | .881 | .180 | .222 |
| *Vibrionimonas* | *r* | .063 | -.114 | .523 | -.144 | -.204 | .208 | -.144 | -.204 | .122 | .309 |
|  | *P* | .846 | .724 | .081 | .656 | .524 | .516 | .656 | .524 | .705 | .328 |
| *Kallotenue* | *r* | -.351 | -.412 | .479 | .063 | .251 | -.255 | .063 | .251 | .096 | .498 |
|  | *P* | .263 | .183 | .115 | .847 | .432 | .423 | .847 | .432 | .766 | .099 |
| *Methylobacter* | *r* | -.704^*^ | -.780^**^ | -.174 | .881^**^ | .694^*^ | -.721^**^ | .881^**^ | .694^*^ | -.045 | -.150 |
|  | *P* | .011 | .003 | .589 | .000 | .012 | .008 | .000 | .012 | .890 | .641 |
| *Blastococcus* | *r* | -.298 | -.037 | .504 | -.201 | .247 | -.012 | -.201 | .247 | -.234 | .026 |
|  | *P* | .347 | .909 | .095 | .531 | .439 | .971 | .531 | .439 | .465 | .936 |
| *Acidobacteria_bacterium_WX27* | *r* | .380 | .450 | -.065 | -.521 | -.239 | .360 | -.521 | -.239 | .111 | .064 |
|  | *P* | .224 | .142 | .840 | .083 | .454 | .250 | .083 | .454 | .732 | .843 |
| *Pseudoxanthomonas* | *r* | -.224 | -.025 | .100 | .086 | .171 | -.288 | .086 | .171 | .428 | .474 |
|  | *P* | .485 | .938 | .758 | .790 | .594 | .363 | .790 | .594 | .165 | .119 |
| *Bradyrhizobium* | *r* | -.088 | -.223 | .234 | .203 | .263 | -.357 | .203 | .263 | -.096 | .146 |
|  | *P* | .786 | .487 | .465 | .526 | .409 | .254 | .526 | .409 | .767 | .652 |
| *unclassified_Blastocatellaceae* | *r* | .630^*^ | .309 | .074 | -.237 | -.236 | .289 | -.237 | -.236 | -.186 | -.364 |
|  | *P* | .028 | .329 | .820 | .458 | .461 | .362 | .458 | .461 | .563 | .244 |
| *Lysobacter* | *r* | .260 | .497 | .008 | -.633^*^ | -.420 | .347 | -.633^*^ | -.420 | .371 | .553 |
|  | *P* | .415 | .101 | .981 | .027 | .174 | .269 | .027 | .174 | .235 | .062 |
| *Leptonema* | *r* | .468 | .261 | .131 | .035 | -.296 | .280 | .035 | -.296 | -.553 | -.655^*^ |
|  | *P* | .125 | .413 | .685 | .914 | .350 | .378 | .914 | .350 | .062 | .021 |
| *Altererythrobacter* | *r* | .043 | .041 | .006 | -.292 | -.097 | .016 | -.292 | -.097 | .519 | .793^**^ |
|  | *P* | .895 | .899 | .984 | .357 | .764 | .961 | .357 | .764 | .084 | .002 |
| *Luteitalea* | *r* | .583^*^ | .665^*^ | -.124 | -.417 | -.526 | .442 | -.417 | -.526 | .039 | .053 |
|  | *P* | .047 | .018 | .701 | .178 | .079 | .150 | .178 | .079 | .903 | .870 |
| *Hydrogenophaga* | *r* | .665^*^ | .291 | -.143 | -.206 | -.388 | .272 | -.206 | -.388 | -.227 | -.099 |
|  | *P* | .018 | .359 | .658 | .520 | .212 | .393 | .520 | .212 | .479 | .759 |
| *Cupriavidus* | *r* | -.273 | -.424 | -.465 | .409 | .220 | -.299 | .409 | .220 | -.260 | -.041 |
|  | *P* | .390 | .170 | .128 | .187 | .492 | .345 | .187 | .492 | .414 | .899 |
| *Brevundimonas* | *r* | -.145 | .224 | -.086 | -.225 | -.108 | .037 | -.225 | -.108 | .698^*^ | .558 |
|  | *P* | .653 | .485 | .789 | .481 | .738 | .909 | .481 | .738 | .012 | .059 |
| *Tellurimicrobium* | *r* | -.680^*^ | -.533 | .253 | .213 | .600^*^ | -.576^*^ | .213 | .600^*^ | .229 | .436 |
|  | *P* | .015 | .074 | .427 | .507 | .039 | .050 | .507 | .039 | .474 | .157 |

Table S3 Cd concentration and content in azolla.

| Treatment | Dry mass/g | Cd concentration /mg L^-1^ | Total Cd content / μg |
| --- | --- | --- | --- |
| T1 | 5.36±0.34 | 0.90±0.10 | 4.84±0.81 |

Table S4 Primer sequence details used for Real-time RT-PCR analysis

| Target | Primer sequence (5´-3´) | Annealing procedure |
| --- | --- | --- |
| *OsNramp5* | F-GCCTTGGTGCTATCGAGGAA  R-TACAGGAAGAACCTGCACCC | 30 s denaturation at 95℃, and then 40 cycles of 15 s at 95℃,  30 s at 60℃ |
| *OsHMA3* | F-CTGGCTCTGGTGATGCTTGT  R-TGAAGATCCCCATCCTCGCA |  |
| GAPDH | F-CCTTTTGTAAGGAGAAAGGAG CAAC  R-ATGGCTCCTCCCAAGCAATC |  |


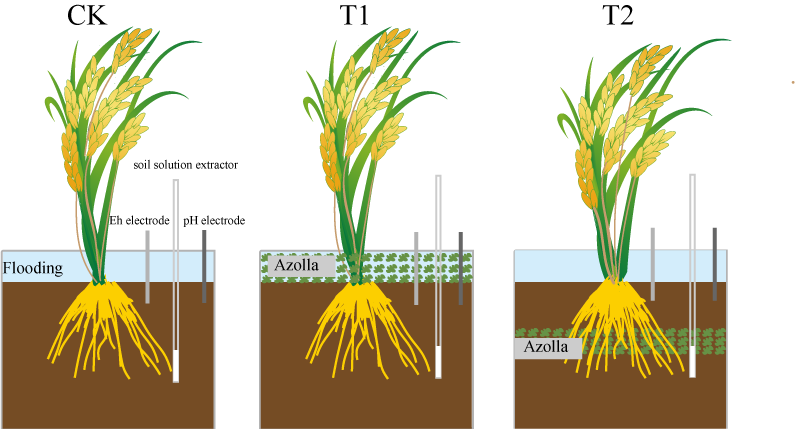


Fig. S1 The diagram of treatments. Treatments: no azolla (CK), azolla culturing in the water layer (T1), azolla incorporated into soil (T2). This image was created using Adobe Illustrator CC 2018 (22.0.0, 64-bit).
